# A Tale of Two Conditions: 50 Years of Chronic Illness and Cancer From a Nursing Perspective

**DOI:** 10.1111/jan.70441

**Published:** 2025-12-12

**Authors:** Sally Thorne

**Affiliations:** ^1^ School of Nursing University of British Columbia Vancouver British Columbia Canada

**Keywords:** assisted dying, cancer, chronic disease, chronic illness, chronicity, end‐of‐life, health care system, health inequity, health policy

## Abstract

**Introduction:**

In this commentary, I consider the disparity between the care we see as a necessity for cancer patients and the lack of care we afford to many persons with chronic disease.

**Background:**

I ground my arguments in my own clinical practice and research experiences over a 50‐year period augmented by reference to available literature sources.

**Data Sources:**

In tracing developments within the fields of cancer and chronic illness care, I draw on my own research and that of others.

**Overview of the Issue:**

Although chronic illness has long been recognised as causing the majority of the burden on our health care systems and as a significant source of suffering in our society, it has not attracted the level of enthusiasm from researchers, policy makers, and health care systems that we have seen in the context of other diseases such as cancer. Nurses have an intimate knowledge of the suffering occasioned by chronic illness; however, it has been difficult for nursing to mobilise coordinated action in prioritising a re‐balance of health systems to better serve those with chronic conditions.

**Findings:**

The advent of medical assistance in dying in Canada has shed a spotlight on the implications of the discrepancy between our prioritising patient need in the care and support of patients with conditions such as cancer, in contrast with the supports and services we make available to those with chronic conditions.

**Discussion:**

Although nursing intimately engages with the burden of chronic illness, it has not mobilized coherent advocacy toward strengthening our societal commitment to this aspect of our care systems.

**Conclusion:**

There is an opportunity for nursing to make a meaningful difference in a fundamental health care system inequity if we can come to understand that chronic illness is as deserving of our collective research, practice change, and policy attention as is cancer.

**Patient or Public Contribution:**

No patient or public contribution.

## What Does This Paper Contribute to the Wider Global Clinical Community?

- While systems of care differ around the world, it is arguable that chronic conditions have been less well served than have many other kinds of diseases such as cancer.
- Unmet need among those with chronic conditions represents a burden on our health care systems, but one that nursing may not have prioritised as a major population health concern.
- The introduction of a new health care system change such as assisted dying can shed a powerful spotlight on system deficiencies, including wide gaps in care that have otherwise been normalised.

## 1 Introduction to Key Moments in the Past 50 Years

I have vivid memories of my time as a nursing student in a hospital program (RN diploma 1972) learning what it was to care for patients with a wide range of conditions. There was an excitement that communicable diseases such as polio were becoming better controlled through scientific advances toward an increasing range of vaccination and antibiotic options. Wound care and infection management were high priority concerns in the clinical context because we could make a meaningful difference to outcomes. Cancer was still greatly feared at that time, as survival rates were considerably lower than they became in subsequent decades (Quaresma et al. 2015). In addition to surgery, which was the primary treatment modality, great advances were being made in both chemo and radiation therapy.

As a new graduate, my first hospital post was in ‘general medicine.’ It would not have been my first choice of patient population at the time but, as it turned out, that unit gave me a fascinating glimpse into the social context surrounding the lives and care options for people with different diseases, including the attitudes of those caring for them. Patients who were potentially curable, or those whose conditions made them candidates for new and exciting interventions (such as dialysis) were in a position of privilege, while those who were considered more ‘chronic’ were understood as somehow less deserving of space and attention within the hospital setting (Lipsitt 1970; Rubin and Davies 1975; Stimson 1974; Whitney 1981).

A decade later, in graduate school, I had the opportunity to critically reflect on the concept of chronicity. Although the idea had already been debated and discounted in sociological circles (Burnham 2013), Talcott Parsons' notion of the ‘sick role’ (Parsons 1951a, 1951b) had considerable appeal for me, not only because it depicted a social ordering of role and responsibility modifications associated with being ill, but more importantly because it helped me understand why persons with chronic conditions were somehow seen as violating role expectations. Over the following years, as my research and clinical inquiry moved back and forth between the fields of chronic illness and cancer care experience from the patient perspective, I remained fascinated by the way in which a highly legitimised and respected disease such as cancer attracted so much research (including that based on philanthropic funding) and investment in clinical services (not only care related to the disease and treatment but also that which was associated with the psychosocial impacts of the condition), while the collection of conditions we might consider under the rubric of ‘chronic’ did not. I am convinced that there is much to be learned from the differential manner in which our mainstream society and health care systems have rendered some health conditions as worthy of our attention, investment, and sustained support, while persons suffering from other kinds of conditions often have so much difficulty accessing the services that might have helped them live their lives. Having now been a registered nurse for over 50 years, I remain concerned that some of these fundamental injustices in terms of how we organise social and health care systems, and who we deem deserving of health care, have yet to be resolved. And I believe that nursing, the profession that sits closest to the intricacies of everyday human experience in the midst of these various conditions, is still ideally positioned to take a lead in witnessing this disparity, speaking ‘truth to power’, and meaningfully advocating for a more equitable and fair approach to those who are ill.

### 1.1 The Social Context of Chronicity

When I think of chronic illness, I include in that configuration a wide range of disease conditions that are socially legitimized (once official diagnosis has been achieved (Deyo et al. 2015)), such as multiple sclerosis, diabetes, or arthritis, and also an assortment of conditions for which there may be no known aetiology or established biomarkers to support a formal diagnosis or a recognized approach to intervention. Some of these conditions have achieved the status of a name based on symptomatology (e.g., myalgic encephalomyelitis, chronic fatigue syndrome, fibromyalgia); others remain as mysterious or undiagnosed chronic conditions (Montano et al. 2022).

In my early chronic illness research, I focused on trying to understand patient experience through the lens of their perspectives on health care relationships and their communications with their health care professionals. What became immediately clear by virtue of these studies was that persons with chronic conditions had very different encounters in health care settings than did those with more acute and episodic conditions (Thorne 1990; Thorne and Robinson 1988). Their experiences were fraught with not being believed when they reported symptoms or concerns, feeling that their health care providers were disinterested or uninformed, and recognising that, unlike those with many other conditions, they could not place their trust in the expertise and good will of care providers, but had to self‐advocate to be taken seriously. My doctoral research, ultimately published in book form because of the complexity of what I wanted to say about these system conditions (Thorne 1993), framed the challenge as one of struggling for recognition (as formal diagnosis was often elusive or delayed by months or even many years after symptom onset), working out the social and health care implications associated with whether the disease was visible (by virtue of altered appearance, mobility, or bodily behaviours), whether they could or could not pass for ‘normal’, and whether what afflicted them was relatively stable or fluctuating over time. Each of these circumstances of illness could lead to different confrontations with the health care system. Among these implications were falling through the care system cracks, being dismissed in favour of patients with more evident intervention paths, getting caught in procedural red tape, such as repeated blood work despite knowing that nothing would show up, and being subjected to what they considered ineptitude or arrogance on the part of clinicians who were determined to convince them that their conditions were not real. Although health care providers were invariably positioned as gatekeepers to the services that might help them, patients also began to appreciate that health care was not apportioned on the basis of the rules of operation they had assumed at the outset (having to do with patient need), but rather by what administrators and clinicians deemed legitimate eligibility criteria (such as evidence‐based disease confirmation). They also began to appreciate that financial incentives played a subtle but important role in clinician priorities (including in a publicly funded health care system) and that turf wars between various clinicians and specialties could significantly influence who had access to what. Having immersed myself in accounts from patients across a very wide range of diagnostic conditions and life situations, I became profoundly convinced that our systems were generally not serving them in the manner that we were serving those with the kinds of conditions for which we preferred to provide care. In my concluding comments, I wrote:

The stories of the chronically ill have enlightened us about the meaning of having ongoing illness in our society. They have horrified us with the distress that a troubled health care system can inflict on such a vulnerable segment of the population. Further, they have convinced us that something is terribly wrong in health care, and that a rather radical reform is urgently required (Thorne 1993, 231).

### 1.2 The Contrasting Social Context of Cancer

Because a majority of cancer patients still died of their condition when my career first began, its association with potential mortality inevitably made it a high‐priority disease across health care systems. Cancer science has advanced dramatically over the previous half‐century in allowing for a better understanding of cumulative genetic and epigenetic changes to normal cells, and in the development of an increasing array of precision medicines and approaches to its management. Cancer is, of course, a wide assortment of different conditions affecting different organ systems or functions characterised by the common feature of uncontrolled proliferation of cells (Brown et al. 2023). As such, the cancer spectrum contains much of the same wide diversity of human experiences and disease manifestations that were so apparent to me in the field of chronic illness. And yet, regardless of those variations, its situatedness within the general collective of ‘cancer’ renders the experience of these diverse conditions dramatically different from the experience of a chronic illness (e.g., Thorne et al. 2005), even, as it turns out, when an effective treatment has rendered that cancer into a chronic condition (Thorne et al. 2013).

When I entered the field of studying human experience with both cancer and chronic illness, the disparity between researcher communities was obvious. Funding that was very difficult to come by for research about patients with chronic illness was readily available if the patient population focus was persons with cancer. There were hundreds of conferences and meetings one could attend to support developing a research career in the cancer field; conferences on chronic illness in a general sense were almost unheard of (although some of the specific conditions, such as diabetes, heart failure, lung disease held such gatherings). Similarly, although there were dozens of scholarly journals focusing on cancer and cancer care at the time when my research began, there were none dedicated to the care of the chronically ill.

The *Journal* *of Chronic Diseases*, published by Elsevier from 1968, was rebranded in 1987 to better fit its mission as the *Journal of Clinical Epidemiology*. The journal *Chronic Illness*, now published by Sage, was not launched until 2005. In the first issue, its editorial team wrote, ‘Central to our manifesto will be the idea that different chronic illnesses are united by often very similar experiences’ (Dowrick et al. 2005). Later in that first year, Halstead Holman acknowledged that, although it was clear that the burden of chronic illness was now consuming the majority of health services, and the role of the patient with a chronic condition was radically different from that of the acute and episodic care patient, modern medicine had not yet adapted in a manner that was conducive to meeting the needs of these patients (Holman 2005). Following an international symposium in 2008 on the theme ‘Managing chronic Illness: Challenges and opportunities for nursing practice and research’ sponsored by the Nethersole School of Nursing at the Chinese University of Hong Kong—a meeting that attracted over 300 nursing scholars from 30 countries and regions—the *Journal of Nursing and Healthcare of Chronic Illness* was launched. In their first editorial for that journal, Co‐Editors Kralik and Lee (2009) acknowledged the increasing threat of chronic illness and the substantial role that nurses played in the management of care for patients with chronic conditions. Sadly, in 2011, after just a few issues, the publisher decided to discontinue the journal, suggesting in their published notification that papers about chronic illness would be welcomed in the *Journal of Clinical Nursing* and the *Journal of Advanced Nursing* (Wiley Publishing Co. 2011). Thus, despite the explosion of cancer‐related publishing venues over these years, it was extraordinarily difficult to gain traction for scholarly journals supporting conversations in nursing and health care specific to the idea of chronic illness. This dramatic difference in both the numbers of journals and the impact factor they continue to attract speaks volumes to the sustained differential in how our society and health care system values cancers over chronic illnesses.

### 1.3 Inequitably Broken Health Care Systems

Although it is common to hear that our health care systems are broken, what is less often examined is the inequitable distribution of this brokenness. For the most part, our health care systems have continued to expand their capacity in support of what is deemed essential treatment for trauma, acute illness, and prioritised diseases such as cancer. Further, they have increasingly recognised that comprehensive care for cancer in particular necessitates considerable supportive care that focuses on the prevention and management of the adverse effects of cancer and its various treatments as well as ‘management of physical and psychological symptoms and side effects across the continuum of the cancer journey from diagnosis through treatment to post‐treatment care’ (Scotté et al. 2023, 2). Supportive care aims to improve the quality of rehabilitation, secondary cancer prevention, survivorship, and end‐of‐life care. No nurse would disagree with the merits of that kind of philosophy with respect to appropriate care.

However, advances in services across many (if not all) of our health care systems have not kept pace as regards expanding chronic disease management and support options or providing chronic illness care. It has been argued that this failure to recognize and adapt to the reality that chronic conditions represent the majority of health care needs is fundamentally what has created the modern crisis in health care (Holman 2020; Chin et al. 2025). Even in national systems with a strong social safety net and a publicly funded health care system, we see increasing disparities between disease sufferers who are more or less well served. We have invested in systems of health designed for primary prevention and tertiary treatment, with much less available to the increasing proportion of society with one or more chronic diseases who are trying to live meaningful lives and manage their conditions as well as possible (Lambert et al. 2021; Schwarz et al. 2022). While patient advocacy has been prominent in keeping cancer in the public eye (despite the diversity of diseases the category includes), a chronic illness advocacy voice has been fragmented by relegation to specific disease conditions and perhaps by the exhaustion of so many of those who are affected. Thus, as the burden of chronic illness continues to deepen, it rarely attracts the kind of public or media attention that we are accustomed to in comparison to gaps in emergency, critical care, or specialty services.

## 2 Future Nursing Challenges

For patients diagnosed with cancer in our health care systems, a pathway to receiving care is generally well supported and straightforward. In contrast, with many chronic conditions it is not. And while we might contemplate a wide range of differences in patient experience throughout the trajectory of being ill with cancer or a chronic condition, changes in the context of end‐of‐life care are starting to sharpen our focus on the impact of this difference. By examining what has come to light in the advent of these expanded options and their implementation across the various conditions, we can start to see a critical need for policy developments shaped by the kind of knowledge that arises from a distinctive nursing perspective.

### 2.1 A New Spotlight on the Implications of Chronic Illness Care Gaps

In Canada, where I conduct my research, the extent of this disparity in patient support has become vividly apparent in the wake of new legislation in 2016 permitting Medical Assistance in Dying (MAID). This option became available following a series of public debates and court challenges pertaining to the rights of persons who have a ‘grievous and irremediable medical condition’ causing ‘enduring and intolerable suffering’ and who wish assistance to end their life (Library of Parliament 2016). Essentially, many people who have lived their lives with a sense of choice and autonomy also wish the same in the context of life's conclusion. In 2021, the law was further amended to permit MAID in cases of persons who, although not facing a reasonably foreseeable natural death, had a medical condition from which they were experiencing a comparable level of enduring and intolerable suffering (Mokosak et al. 2025).

It is estimated that over 200 million people worldwide now have access to some form of legally sanctioned voluntary assistance in dying (Sillitti et al. 2025), and this access is rapidly expanding as new countries consider legislative changes in this regard (Colombo and Dalla‐Zuanna 2024). The introduction of a new end‐of‐life option of the enormity of MAID creates a seismic shift in the landscape of health care. When MAID first became available to Canadians, and in a pattern that has continued to the present day, the majority (> 70%) of requests for MAID are from patients with cancer (Thabet et al. 2023; Watson et al. 2022). Much of the early alarm expressed with respect to the newly implemented legislative option had to do with concerns that the availability of MAID would take the place of offering high‐quality palliative care to dying patients. However, as the most recent report from Health Canada, the federal oversight body, makes clear, there is no evidence that health care resources have been diverted from palliative services to MAID care; rather, it seems apparent that palliative care services have expanded significantly across the system since the advent of MAID, particularly in relation to community‐based supports, and that an increasing proportion (74%) of all persons accessing MAID have also had access to these palliative care supports during the months prior to their death (Health Canada 2025). In this context, it seems likely that these early concerns actually opened up a path toward significant advocacy and system changes across the country with respect to expansions in the number and nature of available palliative care services and in the improved access patients had to them (Dorman et al. 2025; Downar et al. 2025).

Once the 2021 legislation was enacted opening access to MAID for persons who did not have a reasonably foreseeable death, much of the critical concern shifted quickly to spotlight the circumstances associated with access to care for chronic conditions in our country. Here, MAID legislation explicitly included the safeguard that these individuals had to demonstrate to their assessors (in our context, both physicians and nurse practitioners are eligible to assess for MAID) ‘enduring and intolerable physical or psychological suffering that cannot be alleviated under conditions the person considers acceptable’ (Department of Justice 2024). This wording requires those assessing such patients for MAID eligibility to make and document an explicit determination as to the options for managing their condition and relieving suffering to which that individual had access over time, as well as the reasons any available options might not have been feasible or desirable for that person. Early research with this patient population has made it apparent to the assessor community that access to specialty services such as pain clinics has been woefully inadequate, with untenably long wait lists, and that many chronically ill persons have been unable to successfully navigate their way through to services that might have alleviated some of their enduring suffering (Wiebe and Kelly 2023). It has also become apparent that many of the individuals seeking MAID for a chronic condition have not had the benefit of robust primary care relationships that could have provided holistic care in the context of health care encounters of sufficient time to provide such care (Pesut et al. 2024).

Under such circumstances, we can see a high degree of moral complexity in the processes by which we oversee access to MAID (Pesut and Thorne 2025). Some would argue that such services ought to be equitably available to persons whose conditions are chronic, in that they deserve at least as much dignity of choice around the options for their end‐of‐life as we now offer to persons with life‐limiting conditions such as cancer. Others would argue that, because we know that chronic illness services have been generally inadequate in comparison, and that many persons who have sought support for their chronic condition over time have lost trust in the health care system overall, these persons have been rendered vulnerable by society and ought to be deprived access to this option for their own protection (Downar et al. 2023).

### 2.2 Where a Powerful Nursing Voice Is Needed

The nursing profession understands that chronic conditions, especially without fulsome social and health care supports, can seriously erode quality of life and cause enduring suffering, including in many instances a painful downward social spiral, with loss of gainful employment, relationships, and opportunities. Nursing also has an abiding sense of itself as an agent for social justice and an advocate for those who are affected by societal inequities (Fowler 2024). And although our scholarly literature contains a lively and robust discourse on the health equity impacts of a wide range of social determinants of health, we have less often paid thoughtful attention to this issue of chronicity as a category of unjustifiable disparity.

Having observed the health‐care seeking experiences of persons with both cancer and chronic conditions over the course of a career, I see nursing as having normalized the inevitability of these two distinct illness trajectories with respect to where our care systems focus attention. In many ways, I believe we may have uncritically acceded to the prevailing ideology that, although cancer patients are deserving of the absolute best that our science and our care systems can offer, those affected by chronic conditions are not, especially when resources are constrained (and of course they always are). The power of this ideology was made apparent in the HIV/AIDS community when, with the advent of anti‐retroviral agents (across much of the global north at least), the public face of that infection changed from being a death sentence to it being a chronic condition. Although that rapid shift certainly occasioned a sense of relief, it also generated a fear among those affected that, without the urgency of imminent mortality, much of society would lose interest (Deeks et al. 2013).

Nursing has always been closely positioned to the sorrow, loss, pain and distress that chronic illness can entail for patients and their families, and many nurses have shared in the frustration of their patients that services so readily accessible to those with ‘non‐chronic’ conditions may be completely beyond reach in the case of chronicity. Our systems have proven to be effective gatekeepers in deciding who among the suffering deserves our utmost attention and who we can leave to fend for themselves. But we haven't yet found a way to take this acuity/chronicity disparity very seriously in the sense of making it an urgent and pressing professional policy agenda.

It is ironic that MAID seems to have become a trigger for societal awakening with regard to unjustifiable gaps in who and what our health care system serves. However, Canada's recognition that assisted death should never be a default option in the absence of appropriate palliative care seems to have created a widespread call to action to address those gaps. Now that assisted death has become a potential option for those with chronic conditions, it is equally imperative that offering MAID must never be society's default option to manage an inadequate system response to the burden of chronic disease. Nursing, with its intimate relationship to chronic illness experience, is ideally positioned to capitalize on the spotlight that MAID is shining on the general societal attitude toward the chronically ill as a fundamental care disparity and lead that charge toward rectifying it.

## 3 Conclusion

As MAID becomes an increasingly important driver of our health care system conscience, Canadian nurses will be called upon—whether formally in the context of a MAID assessment process or informally in the context of their advocacy for patients—to demonstrate that our systems have given serious consideration to all of the reasonable and available means to relieve our patients' suffering (Pesut et al. 2025). And if we cannot do this, including making strong policy arguments for improved chronic illness services, we will need to accept that this failure may have implications at the level of life or death.

While MAID is not yet legal in many of the nursing world's jurisdictions, there is a reasonable probability that it may become so in the coming decades. The inevitable manner in which it exposes major inequities within our care systems with respect to chronic disease care should be a concern to all nurses. Nurses know the relentless suffering that persons with chronic conditions may endure, as well as the disproportionate barriers they can face to obtaining relief from that suffering. In addition to that knowledge, we have the strength of numbers and the trust of populations. If we can find a way to take chronic illness seriously, to own our expertise in relation to its impact on the lives of those affected, we can begin to build coherent strategies toward shifting the balance and—at last—steering our health care systems and societies toward greater responsibility in meeting those complex needs. It should have been nursing that took a lead in bringing attention to the impact of a widespread preference for health care oriented to acuity over chronicity. And perhaps it still can be.

## Funding

The author has nothing to report.

## Ethics Statement

The author has nothing to report.

## Consent

The author has nothing to report.

## Conflicts of Interest

The author declares no conflicts of interest.

## Data Availability

The author has nothing to report.
